# A real-world disproportionality analysis of Rucaparib: Post-marketing Pharmacovigilance Data

**DOI:** 10.1186/s12885-023-11201-w

**Published:** 2023-08-11

**Authors:** Qilin Zhang, Yiling Ding, Yamin Shu, Jing Chen

**Affiliations:** 1 Department of Pharmacy, Union Hospital, Tongji Medical College, Huazhong University of Science and Technology, Wuhan, 430022 China; 2 Graduate School of Pharmaceutical Sciences, University of Tokyo, Tokyo, 113-0033 Japan; 3 Department of Pharmacy, Tongji Hospital, Tongji Medical College, Huazhong University of Science and Technology, No.1095 Jiefang Avenue, Wuhan, 430030 China

**Keywords:** Adverse event, Data mining, Disproportionality analysis, FAERS, Pharmacovigilance, Rucaparib

## Abstract

**Background:**

Rucaparib has been approved for the maintenance treatment of adult patients with recurrent epithelial ovarian, fallopian tube, or primary peritoneal cancer. However, the long-term safety of rucaparib in large sample population was unknown. The presented study aimed to evaluate rucaparib-associated adverse events (AEs) according to the real-world pharmacovigilance database.

**Methods:**

Disproportionality analysis was conducted to assess the association between rucaparib and its AEs. Data were collected from the international pharmacovigilance database of US FDA Adverse Event Reporting System (FAERS) between January 2017 and June 2022. The characteristics of rucaparib-related AEs, and the onset time were further analyzed.

**Results:**

A total of 9,296,694 AE reports were recorded in the FAERS during the study period, among which 7,087 reports were associated with rucaparib. About 135 rucaparib-related AE signals in 15 system organ class (SOCs) were identified. The most common AEs included anaemia, thrombocytopenia, nausea, vomiting, fatigue, blood creatinine increase, alanine aminotransferase increase, and aspartate aminotransferase increase, which were listed in the label for rucaparib. Of note, 21 new and unexpected significant AEs that off-label were also found in our study, such as preferred term (PTs) of intestinal obstruction, gastrooesophageal reflux disease, blood iron decreased, dehydration, and hypersomnia. The median onset time of rucaparib-related AEs was 12 days (interquartile range [IQR] 1–62 days), and had early failure types.

**Conclusion:**

Our study demonstrated potential new AEs of rucaparib, and further studies were expected to confirm the results.

**Supplementary Information:**

The online version contains supplementary material available at 10.1186/s12885-023-11201-w.

## Introduction

As one of the third most common gynecologic malignancy in the world, ovarian cancer remains an almost uniformly fatal disease, because more than 70% of patients will relapse within the first 5 years [1–3]. Studies have reported that treatment with Poly (adenosine diphosphate-ribose) polymerase (PARP) inhibitors (PARPis) is one of the latest achievements in the study of recurrent ovarian cancer [4, 5]. Rucaparib has been proved to be a selective inhibitor of PARP enzymes such as PARP-1 and PARP-2, which can induce synthetic lethality in cancer cells. It can exert efficacy both in BRCA-mutated patients who can not tolerate further platinum-based chemotherapy and those who respond (completely or partially) to platinum-based chemotherapy independent of the BRCA status [6].

It has been reported that rucaparib induced an overall response rate of 54% and a median duration of response of 9.2 months in relapsed, platinum-sensitive high-grade ovarian carcinoma patients [7]. In an ARIEL 3 study, patients with recurrent platinum-sensitive ovarian cancer who had at least 2 prior lines of chemotherapy and had a complete or partial response to the last platinum-based treatment were randomized to receive rucaparib or placebo maintenance. The median progression-free survival (PFS) for patients with BRCA-mutant carcinomas was 16.6 months in the rucaparib group and 5.4 months in the placebo group (hazard ratio [HR], 0.23; 95% CI, 0.16–0.34; *P* < 0.0001) [8]. In the instructions issued by FDA in 2022, it has been approved for the maintenance treatment of adult patients with recurrent epithelial ovarian, fallopian tube, or primary peritoneal cancer who are in a complete or partial response to platinum-based chemotherapy. Rucaparib is nowadays widely used for the maintenance therapy for patients with recurrent ovarian cancer with BRCA1 and BRCA2 mutations, and metastatic breast cancer, prostate cancer or pancreatic cancer [9–12].

The product description of rucaparib and its early evaluation of post-marketing safety indicated that the most common adverse drug reactions (ADRs) were fatigue, vomiting, diarrhea, nausea, constipation, aspartate aminotransferase (AST)/alanine aminotransferase (ALT) elevation, anemia, thrombocytopenia, neutropenia, rash, abdominal pain, and dyspnea, etc. With the increasing use of rucaparib, some infrequent adverse events (AEs) begin to occur, such as intestinal obstruction, vertigo, dehydration and photosensitivity, etc. Although some safety studies on rucaparib have been reported in several clinical trials and meta-analyses, or systematic reviews [13–16], systematic research on AE signals related to rucaparib based on large international and real-world databases is still lacking.

As a free and open spontaneous reporting system, the Food and Drug Administration Adverse Event Reporting System (FAERS) is now widely used to evaluate the post-marketing safety of drugs. In the present study, the data mining of FAERS is used to detect and analyze the signals of rucaparib-related AEs from the first quarter of 2017 to the second quarter of 2022, so as to explore the situation and general rules of AEs and provide reference for its rational use in clinic.

## Methods

### Data source

This pharmacovigilance study was carried out to analyze rucaparib-associated AEs that were reported in the FAERS database, using data from the first quarter of 2017 (FDA approval of rucaparib) to the second quarter of 2022. The FAERS data were downloaded from the FDA official website, available at https://fis.fda.gov/extensions/FPD-QDE-FAERS/FPD-QDE-FAERS.html. Briefly, the FAERS data files contained seven types of datasets, which were described in detail in our previous study [17]. We managed FAERS data by MySQL 8.0 for further analysis. This study was conducted in accordance with the institutional ethics board of the Union Hospital of Tongji Medical College of Huazhong University of Science and Technology (No. 20,220,185). It also conformed to the tenets of the Declaration of Helsinki. Because this study was an observational study using global open database (FAERS) with anonymized information, not involving treatment intervention or collection of human samples, informed consent was exempted.

### Data extraction and descriptive analysis

Because of the spontaneity of the reports, duplication is inevitable, so the deduplication process should be performed before analysis. We performed the deduplication according to the FDA recommendation [18]. We checked the reports manually to remove the lower PRIMARYID when the CASEID were the same. Moreover, the CASEID which listed in the deleted cases file was further eliminated. We then identified rucaparib-associated cases in both the “drugname” and “prod_ai” columns using “rucaparib” and “RUBRACA” in the “DRUG” files. To improve accuracy, the “role_cod” as primary suspected (PS) was chosen in the DRUG files [19]. All AEs in FAERS are coded by the preferred term (PT) from standardized Medical Dictionary for Regulatory Activities 24.0 (MedDRA 24.0), including five levels, system organ class (SOC), high-level group term (HLGT), high-level term (HLT), preferred term (PT), and lowest-level term (LLT) [20]. Further, a PT can be linked to more than one SOC in MedDRA. Accordingly, MedDRA was used to classify AEs in each report to the corresponding SOC levels in MySQL 8.0. All rucaparib-associated cases extracted from the FAERS database were performed pharmacovigilance analysis according to MedDRA at both SOC and PT levels.

**Fig. 1:**
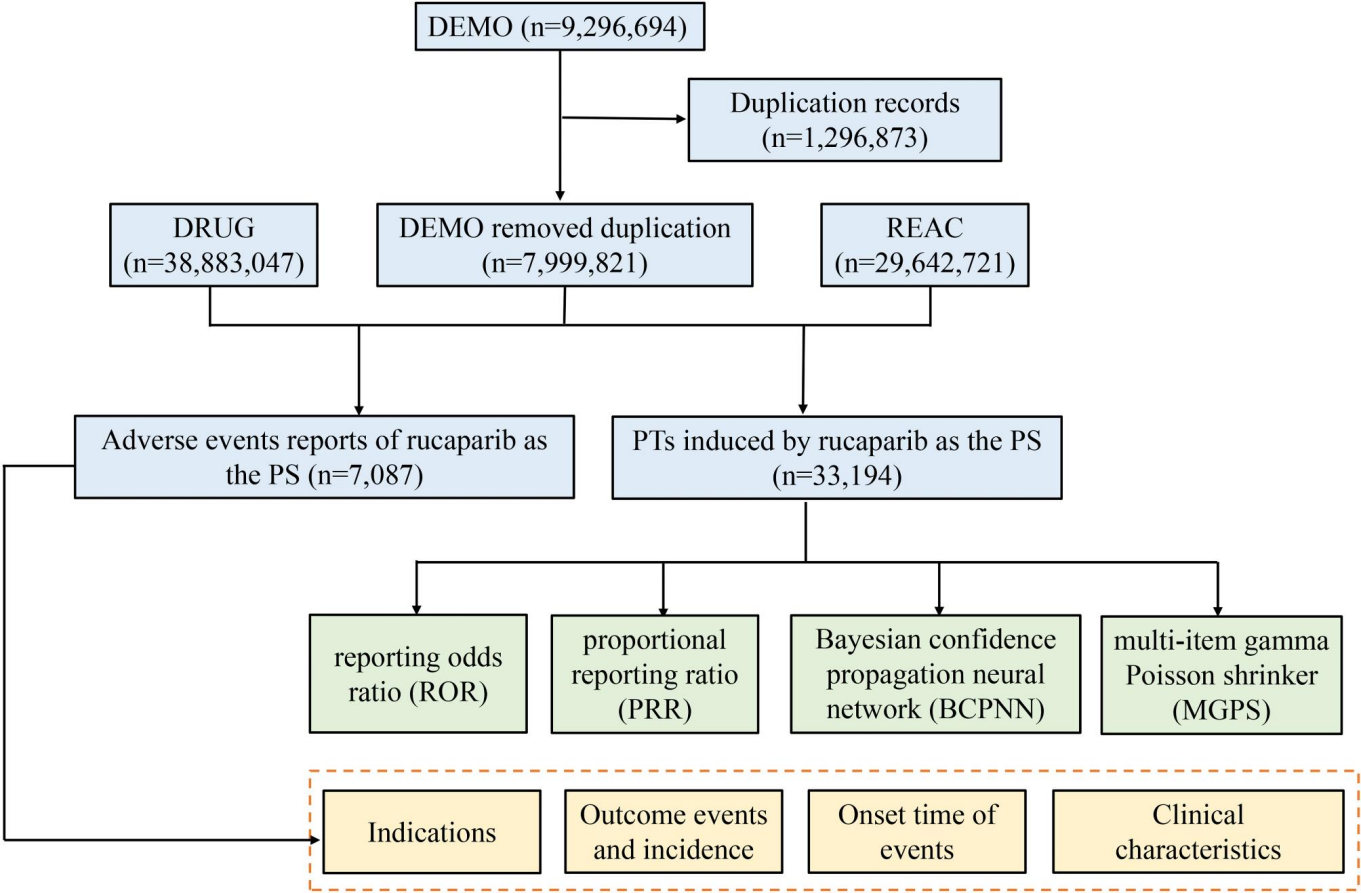
The flow diagram of selecting rucaparib-related AEs from FAERS database

Subsequently, we retrieved and described detailed information, including patient characteristics (gender, age and weight), reporting area, indications, outcomes and reporters, etc. Notably, total serious outcomes may exceed the total number of cases because some cases list more than one serious outcome. For example, a case may go through disability, hospitalization, and then death. The multi-step process of data extraction, processing, and analysis is shown in Fig. 1.

### Data mining

The incidence of AEs cannot be calculated using FAERS database, since we do not know the actual denominators [21]. However, disproportionality analysis, an effective method in pharmacovigilance study, was used to identify signals of disproportionate reporting for AEs related to rucaparib in our study. Both Bayesian and Frequentist methods were employed to explore the association between rucaparib and AEs, by using the reporting odds ratio (ROR), the proportional reporting ratio (PRR), the information component (IC) and the empirical bayes geometric mean (EBGM) [22]. All algorithms were performed to assess whether rucaparib was significantly associated with an AE based on the principles of calculations using a 2 × 2 table. In the present study, AEs were identified as signals when the four algorithms met the criteria outlined above simultaneously. The equations and criteria for the four algorithms are shown in **Supplementary Table 1**.

### Time-to-onset analysis

The time-to-onset (TTO) was calculated using the AE date (EVENT_DT) in the “DEMO” file subtracted the therapy start date (START_DT) in the “THER” file [23]. To ensure the accuracy of calculation, we only used a complete date (YYYYMMDD format) and excluded cases with partial date or without date. We further excluded cases with input errors (EVENT_DT earlier than START_DT). Moreover, TTO analysis was based on medians, quartiles and the Weibull shape parameter (WSP) test. The two parameters (scale parameter α and shape parameter β) were used to describe Weibull distribution, and the shape parameter β was considered and discussed to predict the hazard of the occurrence of AEs over time (i.e. the risk of decrease or increase over time). The definition and criteria for WSP were described in previous literature [24]. All WSP tests were performed using Minitab statistical software (v20.0; Minitab LLC, State College, PA, United States).

## Results

### Descriptive analysis

During the study, 9,296,694 AE reports were retained, among which 7,087 reports were associated with rucaparib after the exclusion of duplicates. The basic characteristics of patients with rucaparib-associated AEs were summarized in Table 1. Females (n = 6,428, 95.51%) accounted for a larger proportion than males (n = 302, 4.49%) due to the specific indications for ovarian and fallopian tube cancer. More than half of the cases were submitted by healthcare professionals (64.94%). Ovarian cancer was the most reported indication (82.80%), followed by fallopian tube cancer (4.22%) and malignant peritoneal neoplasm (3.61%). In terms of age, it was reported more frequently among patients aged 18–65 years than among those older than 65 years (52.45% vs. 47.52%). Hospitalization (37.52%) was the most frequently reported serious outcome, followed by death (13.29%). Most of the AEs were from cases in the US (n = 6,827, 96.33%). Ondansetron, cholecalciferol, gabapentin, vitamins and lorazepam were the top five combination drugs for rucaparib-associated AEs. Nausea, pain, hypertension, anxiety and vomiting were the main comorbidities during rucaparib therapy with 186 (24.16%), 112 (14.55%), 98 (12.73%), 71 (9.22%) and 60 (7.79%) cases, respectively.

**Table 1:** Clinical characteristics of reports with rucaparib from the FAERS database (January 2017 to June 2022)

Characteristics	Rucaparib-induced AE reports (n = 7,087)		
Number of events	Available number, n	Case number, n	Case proportion, %
Gender, n (%)	6,730	-	94.96
Female	-	6,428	95.51
Male	-	302	4.49
Age (years), n (%)	2,944	-	41.54
< 18	-	1	0.03
18 ≤ and ≤ 65	-	1,544	52.45
> 65	-	1,399	47.52
Median (IQR)	-	65 (57–72)	-
Weight (Kg), n (%)	604	-	8.52
< 80	-	407	67.38
80 ≤ and ≤ 100	-	143	23.68
> 100	-	54	8.94
Median (IQR)	-	70.30 (59.38–83.10)	-
Reported countries, n (%)	7,087	-	100.00
US	-	6,827	96.33
Non-US	-	260	3.67
Indications, n (%)	6,704	-	94.60
Ovarian cancer	-	5,551	82.80
Fallopian tube cancer	-	283	4.22
Malignant peritoneal neoplasm	-	242	3.61
Others	-	628	9.37
Combination drugs, n (%)	2,067	-	29.17
Ondansetron	-	553	26.75
Cholecalciferol	-	252	12.19
Gabapentin	-	240	11.61
Vitamins	-	237	11.47
Lorazepam	-	228	11.03
Comorbidities, n (%)	770	-	10.86
Nausea	-	186	24.16
Pain	-	112	14.55
Hypertension	-	98	12.73
Anxiety	-	71	9.22
Vomiting	-	60	7.79
Outcomes, n (%)	7,087	-	100.00
Non-serious Outcome	-	4,334	61.15
Serious Outcome^a^	-	2,753	38.85
Death	-	365	13.29
Life-threatening	-	29	1.05
Hospitalization	-	1,033	37.52
Disability	-	5	0.18
Other serious outcomes	-	1,968	71.49
Time-to-onset (days)	1,968	-	27.77
Median (IQR)	-	12 (1–62)	-
Reporters, n (%)	7,085	-	99.97
Health professional	-	4,601	64.94
Consumer	-	2,484	35.06
Reporting year, n (%)	7087	-	100.00
2022 Q2^b^	-	184	2.60
2021	-	1,158	16.34
2020	-	1,432	20.21
2019	-	1,811	25.55
2018	-	1,321	18.64
2017	-	1,181	16.66

a, Total serious outcomes may exceed the total number of reported cases because some cases list more than one serious outcomes

b, The second quarter of 2022

IQR, interquartile range

### Disproportionality analysis

Signal reports of rucaparib at the SOC level were listed in Table 2. Remarkably, rucaparib-related AEs occurrence were distributed across 27 organ systems. At least one of the four algorithms that met the criteria for significant SOCs related to rucaparib were general disorders and administration site conditions (SOC: 10018065, 4,052), gastrointestinal disorders (SOC: 10017947, 3,387), investigations (SOC: 10022891, 2,435), nervous system disorders (SOC: 10029205, 1,947), metabolism and nutrition disorders (SOC: 10027433, 1,068), cardiac disorders (SOC: 10007541, 980), musculoskeletal and connective tissue disorders (SOC: 10028395, 919), and blood and lymphatic system disorders (SOC: 10005329, 855).

**Table 2:** Signal strength of reports of rucaparib at the System Organ Class (SOC) level in FAERS database

System Organ Class (SOC)	Rucaparib Cases Reporting SOC	ROR (95% two-sided CI)	PRR (χ2)	IC (IC025)	EBGM (EBGM05)
General disorders and administration site conditions	4,052	2.10 (2.00-2.20)^a^	1.47 (995.27)	0.55 (0.50)^a^	1.47 (1.40)
Gastrointestinal disorders	3,387	4.14 (3.96–4.34)^a^	2.64 (4210.2)^a^	1.40 (1.34)^a^	2.64 (2.52)^a^
Investigations	2,435	4.05 (3.86–4.25)^a^	3.00 (3662.82)^a^	1.58 (1.51)^a^	3.00 (2.85)^a^
Injury, poisoning and procedural complications	2,234	1.10 (1.04–1.15)^a^	1.07 (13.27)	0.09 (0.03)^a^	1.07 (1.01)
Nervous system disorders	1,947	1.47 (1.39–1.55)^a^	1.34 (209.47)	0.42 (0.35)^a^	1.34 (1.27)
Neoplasms benign, malignant and unspecified (incl cysts and polyps)	1,470	2.72 (2.56–2.88)^a^	2.36 (1260.98)^a^	1.23 (1.15)^a^	2.36 (2.23)^a^
Metabolism and nutrition disorders	1,068	2.35 (2.20–2.50)^a^	2.14 (699.16)^a^	1.10 (1.00)^a^	2.14 (2.01)^a^
Skin and subcutaneous tissue disorders	1,061	0.92 (0.87–0.99)	0.94 (5.67)	-0.10 (-0.19)	0.94 (0.88)
Respiratory, thoracic and mediastinal disorders	1,056	1.04 (0.97–1.11)	1.03 (1.07)	0.04 (-0.05)	1.03 (0.96)
Cardiac disorders	980	1.29 (1.21–1.39)^a^	1.25 (56.58)	0.32 (0.23)^a^	1.25 (1.17)
Musculoskeletal and connective tissue disorders	919	1.09 (1.02–1.17)^a^	1.08 (6.35)	0.11 (0.01)^a^	1.08 (1.01)
Psychiatric disorders	912	0.98 (0.91–1.05)	0.98 (0.36)	-0.03 (-0.13)	0.98 (0.92)
Blood and lymphatic system disorders	855	2.59 (2.41–2.78)^a^	2.40 (732.09)^a^	1.26 (1.15)^a^	2.39 (2.23)^a^
Vascular disorders	788	0.71 (0.66–0.77)	0.75 (80.12)	-0.42 (-0.53)	0.75 (0.69)
Infections and infestations	736	0.91 (0.84–0.98)	0.92 (6.41)	-0.13 (-0.24)	0.92 (0.85)
Renal and urinary disorders	515	1.05 (0.96–1.15)	1.05 (1.11)	0.06 (-0.07)	1.05 (0.96)
Reproductive system and breast disorders	295	0.89 (0.79-1.00)	0.89 (4.09)	-0.17 (-0.34)	0.89 (0.79)
Surgical and medical procedures	221	0.91 (0.80–1.04)	0.92 (1.77)	-0.13 (-0.33)	0.92 (0.80)
Hepatobiliary disorders	176	0.91 (0.78–1.06)	0.91 (1.47)	-0.14 (-0.36)	0.91 (0.79)
Eye disorders	165	0.54 (0.46–0.63)	0.55 (64.33)	-0.87 (-1.10)	0.55 (0.47)
Immune system disorders	165	0.22 (0.19–0.26)	0.24 (437.51)	-2.06 (-2.28)	0.24 (0.21)
Ear and labyrinth disorders	91	1.01 (0.82–1.24)	1.01 (0.01)	0.00 (-0.31)	1.01 (0.82)
Endocrine disorders	41	0.22 (0.16–0.30)	0.23 (111.7)	-2.15 (-2.61)	0.23 (0.17)
Product issues	36	0.11 (0.08–0.16)	0.12 (248.44)	-3.09 (-3.57)	0.12 (0.08)
Social circumstances	24	0.28 (0.19–0.42)	0.29 (43.33)	-1.82 (-2.41)	0.29 (0.19)
Pregnancy, puerperium and perinatal conditions	5	0.04 (0.02–0.09)	0.04 (120.68)	-4.69 (-5.98)	0.04 (0.02)
Congenital, familial and genetic disorders	4	0.10 (0.04–0.26)	0.10 (33.55)	-3.39 (-4.83)	0.10 (0.04)

^a^ indicates statistically significant signals in algorithm

ROR, reporting odds ratio; CI, confidence interval; PRR, proportional reporting ratio; χ^2^, chi-squared; IC, information component; IC025, the lower limit of 95% CI of the IC; EBGM, empirical Bayesian geometric mean; EBGM05, the lower limit of 95% CI of EBGM.

A total of 135 rucaparib-related AE signals in 15 SOCs were identified in our data analysis. The number of reporting PTs > 20 were shown in Table 3, including 82 PTs and 13 corresponding SOCs, and other PTs ≤ 20 were listed in **Supplementary Table 2**. In the current study, PTs of anaemia, thrombocytopenia, nausea, vomiting, constipation, fatigue, blood creatinine increase, ALT/AST increase, and blood cholesterol increase were detected, which were common AEs listed in the label for rucaparib. Of note, 21 new and unexpected significant AEs that off-label were also found in the present study, such as PTs of intestinal obstruction, gastrooesophageal reflux disease, glomerular filtration rate decreased, blood iron decreased, dehydration, and hypersomnia.

**Table 3:** Signal strength of reports of rucaparib at the Preferred Term (PT) level in FAERS database

SOC	Preferred Terms (PTs)	Rucaparib Cases Reporting PT	ROR (95% two-sided CI)	PRR (χ2)	IC (IC025)	EBGM (EBGM05)
Blood and lymphatic system disorders	Anaemia	472	8.67 (7.90–9.52)	8.16 (2969.23)	3.00 (2.86)	8.11 (7.38)
	Thrombocytopenia	168	4.87 (4.18–5.67)	4.78 (501.92)	2.21 (1.98)	4.76 (4.08)
	Lymphadenopathy^a^	39	3.61 (2.64–4.95)	3.60 (73.10)	1.72 (1.25)	3.59 (2.62)
	Bone marrow failure^a^	38	4.53 (3.29–6.23)	4.51 (103.39)	2.01 (1.54)	4.49 (3.26)
Gastrointestinal disorders	Nausea	2,003	10.93 (10.38–11.52)	8.13 (12877.67)	3.01 (2.93)	8.07 (7.67)
	Vomiting	698	5.35 (4.95–5.79)	4.92 (2216.93)	2.28 (2.17)	4.91 (4.54)
	Diarrhoea	685	3.30 (3.05–3.57)	3.08 (989.66)	1.61 (1.50)	3.07 (2.84)
	Constipation	670	10.39 (9.60-11.26)	9.51 (5107.95)	3.22 (3.10)	9.43 (8.71)
	Abdominal pain	345	5.07 (4.55–5.65)	4.87 (1067.83)	2.26 (2.10)	4.86 (4.36)
	Abdominal pain upper	311	4.96 (4.42–5.56)	4.78 (935.62)	2.23 (2.06)	4.77 (4.26)
	Abdominal discomfort	284	4.82 (4.28–5.43)	4.67 (822.32)	2.19 (2.02)	4.65 (4.13)
	Abdominal distension	186	6.00 (5.19–6.94)	5.87 (750.96)	2.50 (2.29)	5.84 (5.05)
	Dyspepsia	137	4.86 (4.10–5.75)	4.78 (409.84)	2.20 (1.95)	4.77 (4.02)
	Stomatitis	107	5.16 (4.26–6.24)	5.09 (351.41)	2.28 (1.99)	5.07 (4.19)
	Flatulence^a^	103	6.10 (5.02–7.42)	6.03 (430.99)	2.50 (2.22)	6.00 (4.94)
	Dry mouth^a^	90	3.88 (3.15–4.78)	3.84 (189.17)	1.88 (1.57)	3.83 (3.11)
	Intestinal obstruction^a^	84	6.86 (5.53–8.52)	6.80 (413.42)	2.65 (2.33)	6.76 (5.45)
	Gastrooesophageal reflux disease^a^	66	2.74 (2.15–3.49)	2.72 (72.10)	1.39 (1.03)	2.72 (2.13)
	Ascites^a^	61	6.75 (5.24–8.69)	6.70 (294.25)	2.59 (2.21)	6.66 (5.17)
	Small intestinal obstruction^a^	46	11.81 (8.82–15.81)	11.74 (447.55)	3.21 (2.79)	11.63 (8.69)
	Retching	41	6.59 (4.84–8.96)	6.56 (192.11)	2.49 (2.04)	6.52 (4.80)
	Oral pain	38	5.19 (3.77–7.15)	5.17 (127.37)	2.18 (1.71)	5.15 (3.74)
	Abdominal pain lower	25	3.32 (2.24–4.91)	3.31 (40.17)	1.54 (0.96)	3.30 (2.23)
	Eructation	22	4.59 (3.02–6.98)	4.58 (61.35)	1.92 (1.30)	4.57 (3.00)
General disorders and administration site conditions	Fatigue	2,183	11.31 (10.75–11.9)	8.13 (14100.10)	3.01 (2.94)	8.08 (7.69)
	Asthenia	560	5.09 (4.67–5.55)	4.76 (1686.91)	2.24 (2.11)	4.75 (4.36)
	Adverse event	495	18.51 (16.88–20.29)	17.29 (7511.50)	4.04 (3.91)	17.04 (15.54)
	Malaise	344	2.31 (2.08–2.58)	2.25 (243.56)	1.16 (1.00)	2.25 (2.02)
	Drug intolerance	247	6.36 (5.60–7.23)	6.18 (1071.73)	2.58 (2.40)	6.15 (5.41)
	Therapy partial responder	124	32.23 (26.92–38.59)	31.68 (3585.92)	4.63 (4.36)	30.84 (25.76)
	Hernia	31	4.84 (3.40–6.89)	4.82 (93.61)	2.06 (1.54)	4.81 (3.37)
	Early satiety	25	65.04 (43.43–97.40)	64.82 (1485.51)	4.15 (3.56)	61.35 (40.97)
Infections and infestations	Gastroenteritis viral	22	3.92 (2.58–5.96)	3.91(47.55)	1.73 (1.11)	3.90 (2.57)
Investigations	Carbohydrate antigen 125 increased	503	323.52 (292.36–358.00)	300.63 (118633.32)	7.33 (7.19)	237.57 (214.69)
	Platelet count decreased	456	13.98 (12.70-15.37)	13.14 (5080.99)	3.66 (3.52)	13.00 (11.82)
	Haemoglobin decreased	308	10.38 (9.25–11.64)	9.97 (2475.07)	3.26 (3.09)	9.89 (8.82)
	White blood cell count decreased	261	7.22 (6.37–8.17)	6.99 (1337.81)	2.76 (2.58)	6.95 (6.14)
	Weight decreased	248	2.74 (2.42–3.11)	2.68 (264.21)	1.41 (1.22)	2.68 (2.36)
	Blood creatinine increased	234	12.35 (10.83–14.08)	11.97 (2334.98)	3.50 (3.30)	11.86 (10.4)
	Tumour marker increased	231	132.30 (115.23–151.90)	128.02 (26152.22)	6.26 (6.06)	115.07 (100.23)
	Full blood count abnormal	226	18.46 (16.15–21.10)	17.90 (3557.02)	4.03 (3.84)	17.64 (15.44)
	Red blood cell count decreased	215	23.68 (20.64–27.15)	22.99 (4437.42)	4.35 (4.15)	22.55 (19.66)
	Hepatic enzyme increased	151	7.31 (6.22–8.59)	7.17 (799.38)	2.77 (2.53)	7.13 (6.07)
	Laboratory test abnormal	135	11.03 (9.30-13.09)	10.84 (1196.44)	3.32 (3.06)	10.75 (9.06)
	Alanine aminotransferase increased	122	7.62 (6.37–9.12)	7.51 (685.60)	2.81 (2.55)	7.47 (6.24)
	Liver function test increased	119	11.76 (9.80-14.11)	11.58 (1140.47)	3.39 (3.12)	11.47 (9.56)
	Aspartate aminotransferase increased	110	8.51 (7.04–10.27)	8.39 (711.89)	2.95 (2.68)	8.33 (6.9)
	Renal function test abnormal	89	64.08 (51.69–79.43)	63.29 (5167.20)	5.16 (4.85)	59.98 (48.38)
	Blood magnesium decreased^a^	75	26.70 (21.21–33.61)	26.42 (1793.42)	4.26 (3.93)	25.84 (20.53)
	Haematocrit decreased^a^	53	10.15 (7.74–13.32)	10.08 (430.06)	3.07 (2.67)	10.00 (7.62)
	Liver function test abnormal	48	8.67 (6.52–11.53)	8.62 (320.94)	2.86 (2.44)	8.56 (6.44)
	Blood potassium decreased^a^	46	5.04 (3.77–6.74)	5.02 (147.46)	2.17 (1.74)	5.00 (3.74)
	Neutrophil count decreased	44	3.43 (2.55–4.62)	3.42 (75.23)	1.66 (1.23)	3.41 (2.54)
	Blood test abnormal	40	7.78 (5.70-10.63)	7.75 (233.57)	2.69 (2.23)	7.70 (5.64)
	Blood alkaline phosphatase increased	39	6.52 (4.75–8.94)	6.49 (180.17)	2.47 (2.01)	6.46 (4.71)
	Blood cholesterol increased	37	3.27 (2.37–4.52)	3.26 (57.82)	1.58 (1.10)	3.25 (2.35)
	Blood bilirubin increased	33	4.65 (3.30–6.55)	4.63 (93.69)	2.02 (1.51)	4.62 (3.28)
	Glomerular filtration rate decreased^a^	29	7.34 (5.09–10.58)	7.31 (157.04)	2.54 (2.00)	7.27 (5.04)
	Prostatic specific antigen increased^a^	26	5.05 (3.44–7.43)	5.04 (83.86)	2.07 (1.51)	5.02 (3.41)
	Blood urea increased^a^	24	6.51 (4.36–9.74)	6.50 (111.01)	2.35 (1.76)	6.46 (4.32)
	Blood iron decreased^a^	23	5.60 (3.71–8.44)	5.58 (86.16)	2.16 (1.56)	5.56 (3.69)
	Blood sodium decreased	22	4.13 (2.71–6.27)	4.12 (51.73)	1.79 (1.17)	4.10 (2.70)
	Blood urine present	22	3.61 (2.38–5.49)	3.60 (41.31)	1.63 (1.01)	3.60 (2.36)
	Computerised tomogram abnormal^a^	21	41.09 (26.57–63.55)	40.98 (790.34)	3.78 (3.14)	39.57 (25.59)
	Mean cell volume increased^a^	21	23.80 (15.44–36.69)	23.73 (447.88)	3.46 (2.83)	23.26 (15.09)
Metabolism and nutrition disorders	Decreased appetite	684	9.69 (8.95–10.49)	8.85 (4778.46)	3.12 (3.00)	8.79 (8.12)
	Dehydration^a^	166	4.42 (3.79–5.16)	4.34 (427.21)	2.08 (1.85)	4.33 (3.71)
	Hypophagia	76	9.52 (7.59–11.95)	9.43 (568.83)	3.06 (2.73)	9.36 (7.46)
	Feeding disorder	39	5.30 (3.86–7.26)	5.27 (134.52)	2.21 (1.75)	5.25 (3.83)
	Increased appetite^a^	22	4.49 (2.95–6.84)	4.48 (59.35)	1.89 (1.28)	4.47 (2.94)
Musculoskeletal and connective tissue disorders	Bone pain	61	3.22 (2.50–4.14)	3.20 (92.03)	1.60 (1.23)	3.19 (2.48)
Nervous system disorders	Dysgeusia	508	28.27 (25.81–30.98)	26.32 (12125.21)	4.61 (4.48)	25.74 (23.5)
	Taste disorder	229	32.15 (28.13–36.73)	31.14 (6507.63)	4.74 (4.55)	30.33 (26.54)
	Neuropathy peripheral	181	5.68 (4.90–6.59)	5.56 (676.83)	2.43 (2.21)	5.54 (4.78)
	Hypersomnia^a^	39	4.25 (3.10–5.83)	4.23 (96.09)	1.93 (1.47)	4.22 (3.08)
	Parosmia	23	10.46 (6.94–15.79)	10.43 (194.45)	2.84 (2.23)	10.35 (6.86)
Psychiatric disorders	Insomnia	214	2.62 (2.28-3.00)	2.57 (206.87)	1.34 (1.14)	2.56 (2.24)
Renal and urinary disorders	Renal disorder	64	4.01 (3.13–5.13)	3.98 (142.87)	1.90 (1.54)	3.97 (3.11)
Respiratory, thoracic and mediastinal disorders	Dyspnoea exertional	50	3.68 (2.79–4.87)	3.67 (96.77)	1.77 (1.36)	3.66 (2.77)
Skin and subcutaneous tissue disorders	Photosensitivity reaction	156	30.22 (25.74–35.49)	29.58 (4200.75)	4.61 (4.37)	28.85 (24.57)
	Rash pruritic	54	3.43 (2.62–4.48)	3.41 (91.81)	1.68 (1.28)	3.40 (2.60)
Vascular disorders	Hot flush^a^	64	2.81 (2.20–3.59)	2.79 (73.74)	1.42 (1.06)	2.79 (2.18)

^a^ Emerging findings of rucaparib-associated AEs from FAERS database

ROR, reporting odds ratio; CI, confidence interval; PRR, proportional reporting ratio; χ^2^, chi-squared; IC, information component; EBGM, empirical Bayesian geometric mean

Notably, some AEs might unrelated to rucaparib at the PT level were detected (**Supplementary Table 3**), mainly including injury, poisoning and procedural complications (SOC: 10022117), and medical procedures (SOC: 10042613). The SOC of neoplasms benign, malignant and unspecified (incl cysts and polyps) was possibly more associated with disease progression of cancer.

### Time-to-onset of rucaparib-related adverse events

**Fig. 2:**
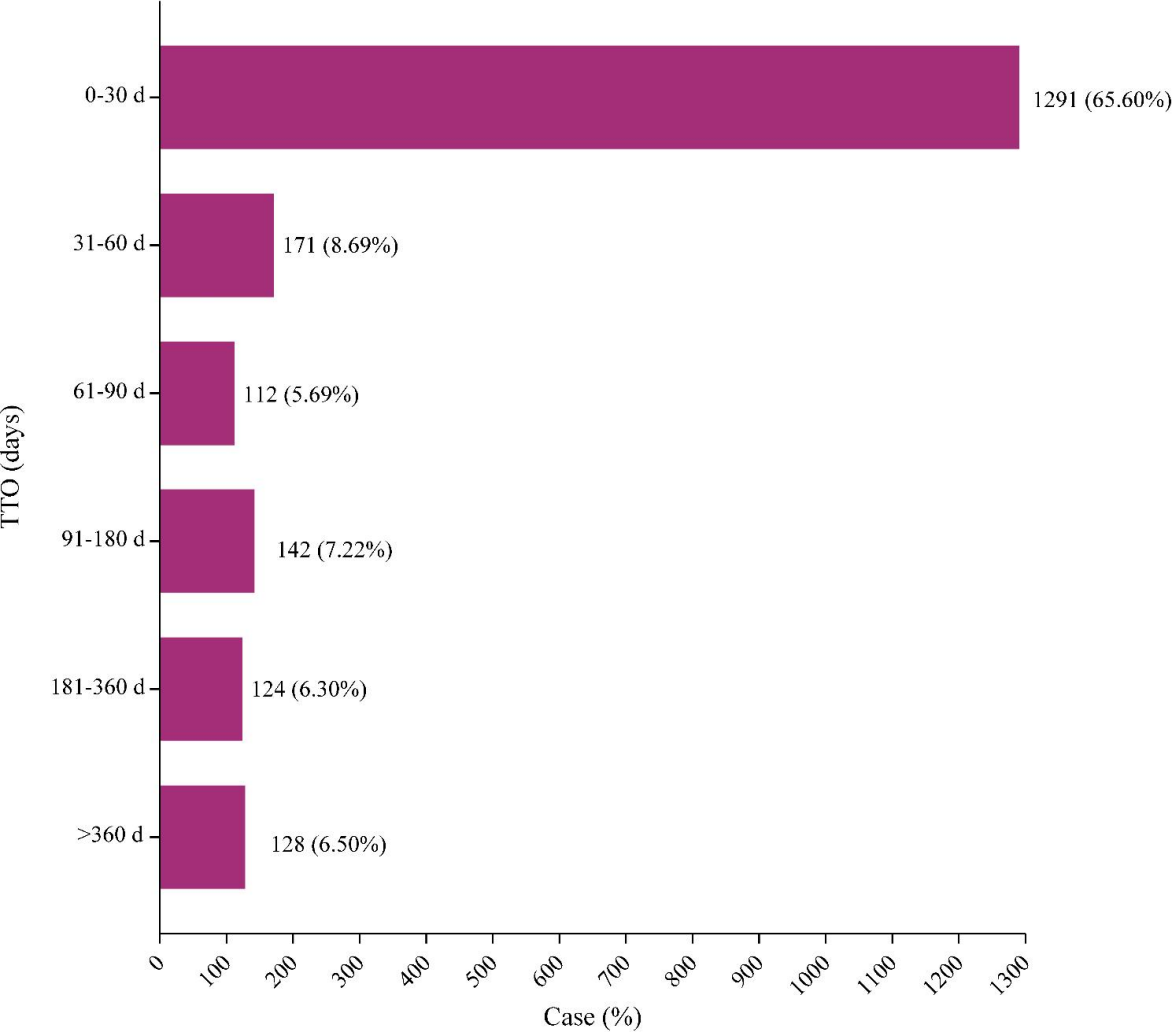
Time-to-onset of rucaparib-related AEs.

**Fig. 3:**
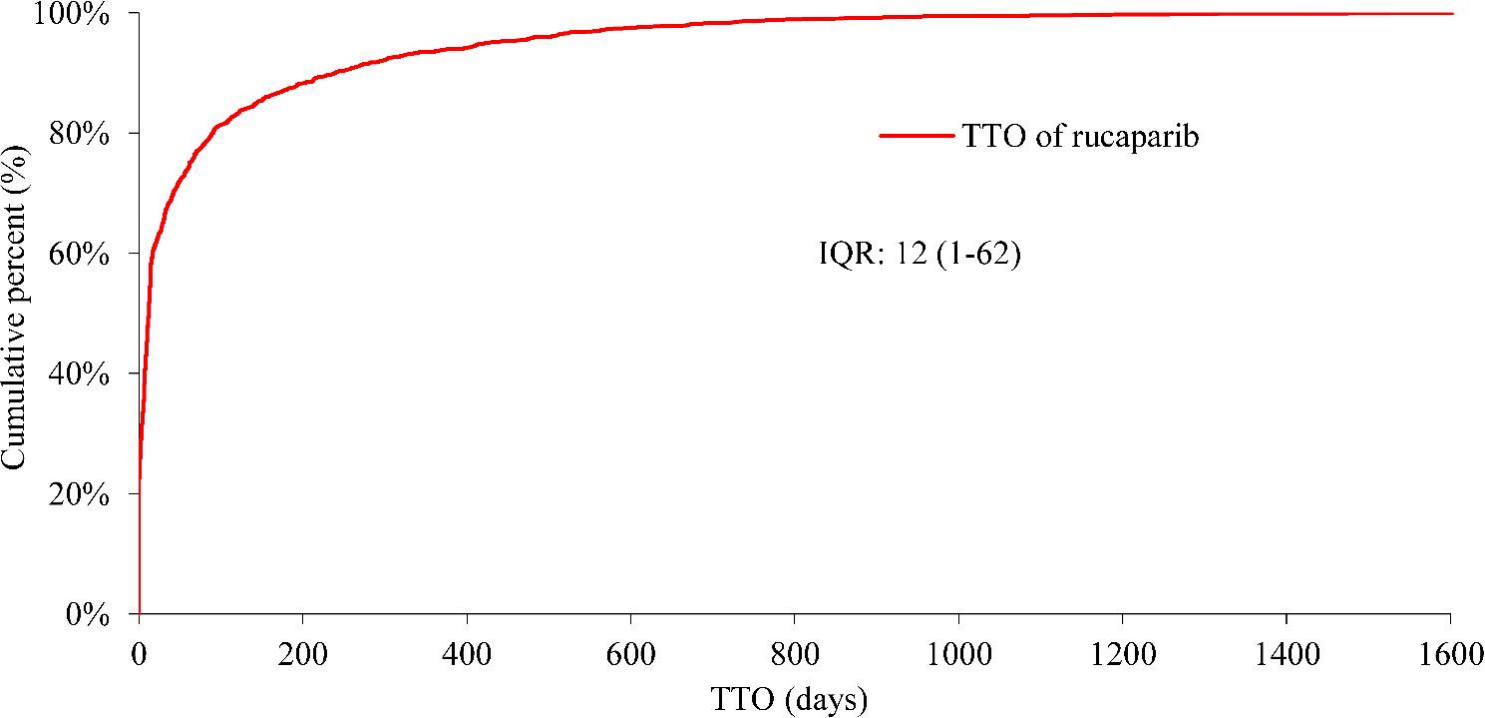
Cumulative distribution curve of TTO.

From January 2017 to June 2022, a total of 1,968 cases in SOC level reported onset time and the median onset time was 12 days (interquartile range [IQR] 1–62 days). Most of the TTO of rucaparib occurred within the first 1 (n = 1,291, 65.60%), 2 (n = 171, 8.69%) and 3 months (n = 112, 5.69%) after initial treatment with rucaparib (Fig. 2), and about 6.50% (n = 128) of AEs occurred 1 year later. Moreover, the cumulative proportion of TTO was shown in Fig. 3. As indicated in Table 4, results demonstrated that the onset time of rucaparib-induced AEs in different SOCs were variable. In the WSP analysis, all shape parameters β and their 95% CI upper limits were less than 1, demonstrating that all AE signals in the SOC level had early failure types.

**Table 4:** Results of time-to-onset analysis for signals in SOC level

SOC	TTO (days)			Weibull distribution				Failure type
	Cases			Scale parameter		Shape parameter		
	n	Median (IQR)	Min-max	α	95% CI	β	95% CI	
All SOCs	1,968	12 (1–62)	0–1,603	16.57	14.07–19.50	0.28	0.27–0.29	Early failure
Blood and lymphatic system disorders	256	27.5 (3-83.5)	0–1,320	31.85	21.24–47.76	0.31	0.28–0.35	Early failure
Cardiac disorders	290	9 (1–31)	0–1,444	11.37	7.65–16.88	0.30	0.27–0.33	Early failure
Ear and labyrinth disorders	24	4.5 (2-28.25)	0-959	18.43	5.08–66.81	0.33	0.24–0.45	Early failure
Endocrine disorders	15	17 (7.5–48.5)	0-618	31.07	8.10-119.12	0.39	0.25–0.60	Early failure
Eye disorders	42	6.5 (2-17.75)	0–1,444	16.54	6.75–40.51	0.35	0.28–0.45	Early failure
Gastrointestinal disorders	985	8 (1–16)	0–1,444	8.72	6.98–10.90	0.29	0.28–0.31	Early failure
General disorders and administration site conditions	1,073	10 (1–34)	0–1,444	12.37	10.04–15.25	0.30	0.28–0.31	Early failure
Hepatobiliary disorders	50	13 (4-40.5)	0-597	24.49	11.06–54.18	0.36	0.29–0.46	Early failure
Immune system disorders	47	10 (2-29.5)	0-777	14.07	6.12–32.31	0.36	0.28–0.45	Early failure
Infections and infestations	203	20 (5–91)	0–1,603	41.42	27.48–62.43	0.35	0.31–0.39	Early failure
Injury, poisoning and procedural complications	643	5 (0-20.5)	0–1,561	3.51	2.40–5.14	0.21	0.20–0.23	Early failure
Metabolism and nutrition disorders	292	8 (1-21.25)	0–1,087	10.17	7.03–14.71	0.32	0.29–0.36	Early failure
Musculoskeletal and connective tissue disorders	217	9 (2–18)	0–1,444	15.85	10.45–24.04	0.33	0.30–0.37	Early failure
Neoplasms benign, malignant and unspecified (incl cysts and polyps)	356	14 (1-120.5)	0-909	23.92	16.03–35.68	0.27	0.25–0.30	Early failure
Nervous system disorders	530	8 (1-28.75)	0–1,444	11.89	8.90–15.90	0.31	0.28–0.33	Early failure
Product issues	8	20.5 (0.75–87.25)	0-511	14.16	0.75-268.97	0.25	0.14–0.45	Early failure
Psychiatric disorders	237	8 (2–31)	0–1,444	12.65	8.33–19.20	0.32	0.28–0.35	Early failure
Renal and urinary disorders	140	14 (4.75–48.25)	0–1,561	28.38	18.11–44.47	0.38	0.33–0.44	Early failure
Reproductive system and breast disorders	72	38.5 (9-157.25)	0-756	69.82	43.78-111.34	0.51	0.43–0.62	Early failure
Respiratory, thoracic and mediastinal disorders	289	12 (3–81)	0–1,603	28.14	19.57–40.46	0.33	0.30–0.36	Early failure
Skin and subcutaneous tissue disorders	256	10 (2-37.5)	0–1,561	15.25	10.17–22.86	0.31	0.28–0.35	Early failure
Surgical and medical procedures	66	51.5 (10-200.5)	0–1,444	70.32	36.45-135.67	0.38	0.31–0.47	Early failure
Vascular disorders	221	8 (1–36)	0–1,444	9.99	6.08–16.43	0.28	0.25–0.31	Early failure

n, number of cases with available time-to-onset; IQR, interquartile range; TTO, Time-to-onset. When TTO, is 0 days, the adverse event occurred within the same day with the therapy

## Discussion

Among the AEs of rucaparib, risk of grade 3–4 were relatively high and grade 3 AEs have been observed in 75% patients [25, 26]. In 58.6% and 9.8% of patients, rucaparib was interrupted and discontinued, respectively and 45.9% of patients were reduced the dose because of treatment-related AEs [27]. A pharmacovigilance approach was used in our study for exploring the relationship between rucaparib and its AEs, so as to evaluate its post-marketing safety. Compared with men (4.49%), women (95.51%) were more likely to occur AEs, which was because rucaparib was mainly used to treat ovarian cancer and breast cancer. When the dose of rucaparib is 600 mg bid, the common AEs are fatigue (12.9%), thrombocytopenia (18.8%), neutropenia (27.1%), anemia (11.8%) and nausea (7.1%), which are included in the drug description, and our research results also confirm it [28, 29].

One of the most common AEs of rucaparib is hematologic toxicity. Among the hematological AEs, anemia was the most frequently reported PARPi-induced AE in a real world study [30], and it could be associated with symptoms that affect patient quality of life (e.g. light-headedness and fatigue). In addition to anemia, other common hematotoxicity of rucaparib include thrombocytopenia, and neutropenia [29, 31]. For grade 2 or higher thrombocytopenia/decreased platelet count, rucaparib is recommended to be interrupted and resumed at a lower dose upon recovery to grade 1 or better. Heparin use is also one of the risk factors for thrombocytopenia, which will be concerned in clinic. For patients with asymptomatic neutropenia/decreased neutrophil count, rucaparib can usually be continued without dose modification. Antibiotic prophylaxis should be initiated in patients with grade 4 afebrile neutropenia, and rucaparib should be remain in use until recovery with a lower dose [32].

As a significant AE of rucaparib, blood creatinine abnormal was reported with signal strength being ROR 10.94 (6.33–18.91), PRR 10.93 (116.11), IC 2.56 (1.76) and EBGM 10.83 (6.27), respectively, in our results. In patients treated with rucaparib, elevated serum creatinine level has been observed. This AE may be due to the inhibition of tubule transporters MATE1, MATE2-K and OCT-2, which resulting in reduced creatinine secretion in proximal tubules [33]. To reduce the risk, renal function monitoring and effective management can be carried out in rucaparib therapy for intervention. Management with dose reduction or treatment delay is necessary.

Although myelodysplastic syndrome (MDS)/acute myeloid leukemia (AML) are rarely observed in patients receiving rucaparib treatment in Phase II (Study 10 and ARIEL 2) and Phase III (ARIEL 3) studies, they are still potentially fatal AEs [7, 27]. MDS and AML are heterogeneous diseases characterized by highly unstable chromosomes, with a variety of potential molecular abnormalities, which are generally believed to be caused by the mechanism of wrong DNA damage repair [34]. PARPis can increase the risk of MDS and AML through DNA damaging reactions, because it can lead to acquired mutations with clonal hematopoiesis in the circulatory system. Furthermore, PARPis may cause off-target epigenetic changes through potential clonal hematopoietic transformations, which can also result in MDS and AML [35]. Our study observed that rucaparib-related bone marrow disorder had a signal strength with ROR 8.81 (4.39–17.67), PRR 8.80 (54.91), IC 2.06 (1.04) and EBGM 8.74 (4.36), respectively. The risk of AEs with MDS/AML usually emerged after long-term treatment, suggesting that caution should be taken when prescribing long-term rucaparib [35]. It is reported that the use of anti-cancer drugs, including alkylating agents, topoisomerase inhibitors, platinum drugs and bevacizumab, will significantly increase the risk of MDS/AML. Therefore, when using rucaparib, it is necessary to avoid the combined use of the above anti-cancer drugs [36].

In the label of rucaparib, rash is one of the ADR reported in ≥ 20% of patients including blister, blood blister, dermatitis, dermatitis contact, eczema, genital rash, palmar-plantar erythrodysaesthesia syndrome, skin lesion, skin exfoliation and urticaria. Our study showed some other related AEs such as nail discolouration, hair growth abnormal, solar dermatitis and onychomadesis. Among them solar dermatitis should be noticed, because this AE had significant signal strength in the skin and subcutaneous tissue disorders of rucaparib in our study. Evaluation of phototoxicity induced by rucaparib has demonstrated that rucaparib can trigger photosensitivity reactions. This phototoxicity can be attributed to photosensitized damage towards main cellular biomolecules (lipids, proteins and DNA) [37].

It is noteworthy that in our analysis, we also found 21 new and unexpected AEs that not mentioned in the drug description including intestinal obstruction, ascites, gastrooesophageal reflux disease, glomerular filtration rate decreased, blood iron decreased, dehydration and hypersomnia. The results of an international, multicenter, open-label phase 2 trial showed that 204 patients who received rucaparib had serious AEs including small intestinal obstruction (10 [5%] of 204 patients), malignant neoplasm progression (10 [5%]), and anaemia (9 [4%]). Grade 1–2 and grade 3 about dehydration was 4.9% (10/204) and 2.9% (6/204), respectively in the same study [7]. Gastrointestinal disorders are the common AEs of rucaparib in our study, including nausea (n = 2,003), vomiting (n = 698), diarrhoea (n = 685) and constipation (n = 670),which are in line with results in the clinical trial [7]. Gastroesophageal reflux has been reported to be causally associated with adiposity, diabetes, smoking and high caffeine consumption, even major depressive disorder [38, 39]. Ascites may be an important AE caused by rucaparib with ROR 6.75 (5.24–8.69), PRR 6.70 (294.25), IC 2.59 (2.21) and EBGM 6.66 (5.17), respectively, which deserves the attention of clinicians. In a study evaluating the pharmacokinetics and safety of rucaparib in patients with advanced solid tumors, the most common treatment-emergent adverse events (TEAEs) observed at the SOC level were gastrointestinal disorders (18.8%), including abdominal pain, ascites, and nausea [40]. Due to the relatively recent introduction of rucaparib, its safety needs to be supported by big data analysis of real-world self-reported AEs.

In terms of the occurrence time of rucaparib-related AEs, our research showed most of them occurred in the early stage of treatment, which was consistent with the results reported [8]. The majority of the AEs occurred within the first 1 month (n = 1,291, 65.60%), 2 months (n = 171, 8.69%) and 3 months (n = 112, 5.69%) after rucaparib treatment, with 12 days of the median onset time. Besides, the WSP test in our study revealed that all rucaparib-associated AEs in SOC level had an early failure type profile, suggesting that the risk of rucaparib-associated AEs increased at an earlier stage of treatment and then gradually decreased over time. But the risk of some fetal AEs including MDS and AML usually emerged after long-term treatment [35]. Therefore, in future clinical studies, rucaparib related AEs required a longer follow-up time for further observation.

Because FAERS database reporting used in our study is voluntary, some of the AE reports may be arbitrary, biased, underreporting, or have incomplete content, etc. which will also affect the results. In addition, although oral administration is convenient and shows favorable compliance with the majority of patients, it may be affected by numerous factors, including food, metabolic enzymes and transporters. These interactions may be associated with serious AEs or may reduce the treatment efficacy of rucaparib, which need continuous attention. Because FAERS data does not grade AEs, only serious and non-serious outcomes caused by AEs were reported. Therefore, we are unable to provide grade classification on each AE. In the data analysis, unmeasured multiple confounding factors including potential DDI, drug combinations and patient comorbidities that may affect AEs were not included. Further research is needed in the future.

## Conclusion

Based on the FAERS database, we assessed the post-marketing safety profiles of rucaparib. Common AEs of anaemia, thrombocytopenia, nausea, blood creatinine increase, and AST/ALT increase were detected. Of note, 21 new and unexpected significant AEs that off-label were also found in the present study. Rucaparib-associated AEs in SOC level had an early failure type profile, suggesting that the risk of rucaparib-associated AEs increased at an earlier stage of treatment and then gradually decreased over time. Our study provides important support for clinical safety studies of rucaparib.

### Electronic supplementary material

Below is the link to the electronic supplementary material.

Supplementary Material 1

## Data Availability

The database used in this study is publicly available in website of https://fis.fda.gov/extensions/FPD-QDE-FAERS/FPD-QDE-FAERS.html.
