# Supplementary material for: A real-world disproportionality analysis of Rucaparib: Post-marketing Pharmacovigilance Data
Source: BMC Cancer. 2023 Aug 11;23:745. doi: 10.1186/s12885-023-11201-w (PMC10416473; doi:10.1186/s12885-023-11201-w)
Supplement: Supplementary file 1 — Supplementary Material 1 [file 12885_2023_11201_MOESM1_ESM.docx]

**Supplementary Table 1. Four major algorithms used for signal detection.**

| Algorithms | Equation | Criteria |
| --- | --- | --- |
| ROR | ROR=ad/b/c | lower limit of 95% CI>1, N≥3 |
|  | 95%CI=e^ln(ROR)±1.96(1/a+1/b+1/c+1/d)^0.5^ |  |
| PRR | PRR=a(c+d)/c/(a+b) | PRR≥2, χ^2^≥4, N≥3 |
|  | χ^2^=[(ad-bc)^2](a+b+c+d)/[(a+b)(c+d)(a+c)(b+d)] |  |
| BCPNN | IC=log_2_a(a+b+c+d)(a+c)(a+b) | IC025>0 |
|  | 95%CI= E(IC) ± 2V(IC)^0.5 |  |
| MGPS | EBGM=a(a+b+c+d)/(a+c)/(a+b) | EBGM05>2 |
|  | 95%CI=e^ln(EBGM)±1.96(1/a+1/b+1/c+1/d)^0.5^ |  |

Equation: a, number of reports containing both the target drug and target adverse drug reaction; b, number of reports containing other adverse drug reaction of the target drug; c, number of reports containing the target adverse drug reaction of other drugs; d, number of reports containing other drugs and other adverse drug reactions. 95%CI, 95% confidence interval; N, the number of reports; χ^2^, chi-squared; IC, information component; IC025, the lower limit of 95% CI of the IC; E(IC), the IC expectations; V(IC), the variance of IC; EBGM, empirical Bayesian geometric mean; EBGM05, the lower limit of 95% CI of EBGM.

**Supplementary Table 2. Signal strength of reports of rucaparib at the Preferred Term (PT) level in FAERS database (n≤20).**

| SOC | Preferred Terms (PTs) | Rucaparib Cases  Reporting PT | ROR  (95% two-sided CI) | PRR (χ2) | IC (IC025) | EBGM (EBGM05) |
| --- | --- | --- | --- | --- | --- | --- |
| Blood and lymphatic system disorders | Blood disorder | 10 | 4.10 (2.20-7.63) | 4.10 (23.32) | 1.54 (0.62) | 4.08 (2.19) |
|  | Bone marrow disorder | 8 | 8.81 (4.39-17.67) | 8.80 (54.91) | 2.06 (1.04) | 8.74 (4.36) |
| Ear and labyrinth disorders | Motion sickness^a^ | 7 | 12.79 (6.07-26.94) | 12.77 (75.12) | 2.17 (1.07) | 12.64 (6.00) |
| Gastrointestinal disorders | Abdominal tenderness | 17 | 12.23 (7.58-19.74) | 12.20 (173.03) | 2.82 (2.12) | 12.08 (7.49) |
|  | Gastrointestinal motility disorder | 12 | 6.37 (3.61-11.24) | 6.36 (53.91) | 2.05 (1.22) | 6.33 (3.59) |
|  | Lip dry^a^ | 9 | 4.81 (2.50-9.25) | 4.80 (26.98) | 1.64 (0.68) | 4.79 (2.49) |
|  | Oral disorder^a^ | 9 | 3.97 (2.06-7.65) | 3.97 (19.94) | 1.46 (0.50) | 3.96 (2.06) |
|  | Oral mucosal blistering^a^ | 9 | 3.90 (2.03-7.52) | 3.90 (19.35) | 1.44 (0.48) | 3.89 (2.02) |
|  | Tongue discolouration^a^ | 9 | 7.84 (4.07-15.11) | 7.83 (53.28) | 2.06 (1.10) | 7.78 (4.04) |
|  | Dyschezia | 7 | 5.18 (2.46-10.89) | 5.18 (23.49) | 1.57 (0.48) | 5.16 (2.45) |
|  | Gastrointestinal sounds abnormal | 7 | 4.89 (2.32-10.27) | 4.88 (21.52) | 1.52 (0.43) | 4.87 (2.31) |
|  | Lip blister^a^ | 7 | 8.03 (3.82-16.89) | 8.02 (42.73) | 1.90 (0.80) | 7.97 (3.79) |
|  | Regurgitation^a^ | 7 | 7.89 (3.75-16.61) | 7.89 (41.81) | 1.89 (0.79) | 7.84 (3.73) |
|  | Chapped lips^a^ | 6 | 4.72 (2.12-10.52) | 4.72 (17.50) | 1.40 (0.22) | 4.70 (2.11) |
|  | Intestinal mass | 6 | 13.18 (5.89-29.47) | 13.17 (66.67) | 2.04 (0.85) | 13.02 (5.82) |
|  | Abdominal mass | 5 | 6.44 (2.67-15.52) | 6.44 (22.84) | 1.49 (0.20) | 6.41 (2.66) |
|  | Large intestinal obstruction^a^ | 5 | 6.01 (2.49-14.47) | 6.01 (20.75) | 1.45 (0.15) | 5.98 (2.48) |
|  | Lip discolouration^a^ | 5 | 13.06 (5.41-31.55) | 13.05 (55.02) | 1.85 (0.55) | 12.92 (5.35) |
| General disorders and administration site conditions | Nodule^a^ | 17 | 3.95 (2.45-6.37) | 3.94 (37.27) | 1.68 (0.97) | 3.93 (2.44) |
|  | Temperature intolerance | 17 | 4.97 (3.09-8.01) | 4.96 (53.57) | 1.94 (1.24) | 4.94 (3.07) |
|  | Thirst^a^ | 16 | 3.31 (2.03-5.41) | 3.31 (25.67) | 1.45 (0.73) | 3.30 (2.02) |
|  | Pelvic mass | 5 | 21.46 (8.85-51.99) | 21.44 (95.62) | 2.01 (0.71) | 21.06 (8.69) |
| Hepatobiliary disorders | Hepatic lesion | 7 | 5.23 (2.49-11.00) | 5.23 (23.83) | 1.58 (0.48) | 5.21 (2.48) |
| Investigations | Red cell distribution width increased | 19 | 10.98 (6.99-17.27) | 10.96 (170.27) | 2.79 (2.12) | 10.86 (6.91) |
|  | Vitamin D decreased | 15 | 5.31 (3.20-8.83) | 5.30 (52.15) | 1.97 (1.22) | 5.28 (3.18) |
|  | White blood cell count abnormal | 14 | 5.45 (3.22-9.22) | 5.44 (50.57) | 1.97 (1.19) | 5.42 (3.21) |
|  | Blood creatinine abnormal | 13 | 10.94 (6.33-18.91) | 10.93 (116.11) | 2.56 (1.76) | 10.83 (6.27) |
|  | Hepatic enzyme abnormal | 12 | 7.29 (4.13-12.87) | 7.28 (64.65) | 2.18 (1.34) | 7.24 (4.10) |
|  | Scan abnormal^a^ | 12 | 96.14 (53.30-173.42) | 95.98 (1039.48) | 3.40 (2.54) | 88.53 (49.08) |
|  | Mean cell haemoglobin increased | 11 | 18.68 (10.29-33.92) | 18.66 (180.82) | 2.78 (1.90) | 18.37 (10.12) |
|  | Positron emission tomogram abnormal^a^ | 10 | 50.42 (26.75-95.04) | 50.35 (463.03) | 3.05 (2.12) | 48.24 (25.59) |
|  | Imaging procedure abnormal^a^ | 9 | 241.98 (117.75-497.28) | 241.67 (1776.43) | 3.11 (2.06) | 199.20 (96.93) |
|  | Platelet count abnormal | 9 | 3.88 (2.01-7.46) | 3.87 (19.12) | 1.43 (0.47) | 3.86 (2.01) |
|  | Thyroid function test abnormal | 9 | 6.54 (3.39-12.6) | 6.53 (41.93) | 1.92 (0.95) | 6.50 (3.37) |
|  | Vitamin B12 decreased^a^ | 9 | 9.50 (4.93-18.31) | 9.49 (67.76) | 2.20 (1.24) | 9.42 (4.88) |
|  | Blood albumin decreased | 8 | 4.22 (2.10-8.44) | 4.21 (19.53) | 1.46 (0.44) | 4.20 (2.10) |
|  | Red blood cell count abnormal | 8 | 14.81 (7.37-29.75) | 14.79 (101.54) | 2.37 (1.34) | 14.61 (7.27) |
|  | Red blood cell count increased | 8 | 7.91 (3.94-15.86) | 7.90 (47.89) | 1.99 (0.96) | 7.85 (3.92) |
|  | Paracentesis | 5 | 19.06 (7.87-46.15) | 19.05 (84.10) | 1.98 (0.68) | 18.75 (7.74) |
| Metabolism and nutrition disorders | Food intolerance | 6 | 4.69 (2.10-10.46) | 4.69 (17.33) | 1.39 (0.21) | 4.67 (2.09) |
| Musculoskeletal and connective tissue disorders | Musculoskeletal chest pain | 17 | 3.55 (2.20-5.72) | 3.54 (30.97) | 1.55 (0.85) | 3.54 (2.20) |
|  | Flank pain | 12 | 4.36 (2.47-7.68) | 4.35 (30.85) | 1.67 (0.84) | 4.34 (2.46) |
| Psychiatric disorders | Initial insomnia | 19 | 7.62 (4.85-11.97) | 7.60 (108.23) | 2.43 (1.77) | 7.56 (4.81) |
| Renal and urinary disorders | Bladder pain^a^ | 5 | 5.11 (2.12-12.31) | 5.11 (16.45) | 1.33 (0.04) | 5.09 (2.11) |
| Respiratory, thoracic and mediastinal disorders | Sinus congestion | 15 | 4.08 (2.45-6.77) | 4.07 (34.62) | 1.68 (0.93) | 4.06 (2.44) |
|  | Upper-airway cough syndrome | 12 | 4.04 (2.29-7.12) | 4.03 (27.30) | 1.59 (0.76) | 4.02 (2.28) |
|  | Hiccups^a^ | 11 | 4.68 (2.59-8.46) | 4.67 (31.61) | 1.71 (0.84) | 4.66 (2.57) |
|  | Paranasal sinus hypersecretion^a^ | 6 | 5.73 (2.57-12.78) | 5.72 (23.28) | 1.55 (0.37) | 5.70 (2.56) |
| Skin and subcutaneous tissue disorders | Nail discolouration^a^ | 12 | 9.12 (5.17-16.11) | 9.11 (85.93) | 2.37 (1.53) | 9.04 (5.12) |
|  | Hair growth abnormal^a^ | 9 | 3.90 (2.02-7.50) | 3.89 (19.29) | 1.44 (0.48) | 3.88 (2.02) |
|  | Solar dermatitis | 7 | 66.41 (30.97-142.37) | 66.34 (425.47) | 2.65 (1.53) | 62.71 (29.25) |
|  | Onychomadesis^a^ | 5 | 4.87 (2.02-11.72) | 4.87 (15.29) | 1.30 (0.01) | 4.85 (2.01) |
| Vascular disorders | Lymphoedema^a^ | 10 | 4.30 (2.31-8.01) | 4.30 (25.20) | 1.58 (0.67) | 4.28 (2.30) |

^a^ Emerging findings of rucaparib-associated AEs from FAERS database.

ROR, reporting odds ratio; CI, confidence interval; PRR, proportional reporting ratio; χ^2^, chi-squared; IC, information component; EBGM, empirical Bayesian geometric mean.

**Supplementary Table 3. Signal strength of reports unrelated to rucaparib at the Preferred Term (PT) level in FAERS database.**

| SOC | Preferred Terms (PTs) | Rucaparib Cases  Reporting PT | ROR  (95% two-sided CI) | PRR (χ2) | IC (IC025) | EBGM (EBGM05) |
| --- | --- | --- | --- | --- | --- | --- |
| Injury, poisoning and procedural complications | Product dose omission issue | 969 | 5.39 (5.03-5.77) | 4.79 (2976.56) | 2.25 (2.15) | 4.77 (4.46) |
|  | Underdose | 229 | 7.54 (6.61-8.61) | 7.33 (1249.09) | 2.82 (2.63) | 7.29 ( 6.39) |
|  | Prescribed underdose | 154 | 16.73 (14.24-19.65) | 16.39 (2196.25) | 3.87 (3.63) | 16.17 (13.77) |
|  | Accidental underdose | 91 | 19.58 (15.90-24.12) | 19.34 (1557.21) | 3.98 (3.67) | 19.03 (15.45) |
|  | Sunburn | 67 | 33.46 (26.22-42.71) | 33.16 (2030.4) | 4.44 (4.08) | 32.24 (25.26) |
|  | Product dose omission in error | 58 | 12.59 (9.71-16.32) | 12.49 (606.78) | 3.35 (2.97) | 12.36 (9.54) |
|  | Intentional underdose | 34 | 9.95 (7.09-13.95) | 9.90 (269.91) | 2.93 (2.43) | 9.83 (7.00) |
|  | Gastrointestinal stoma complication | 5 | 8.80 (3.65-21.23) | 8.80 (34.29) | 1.67 (0.37) | 8.74 (3.62) |
|  | Labelled drug-drug interaction issue | 4 | 15.40 (5.74-41.33) | 15.40 (53.12) | 1.66 (0.21) | 15.20 (5.67) |
| Neoplasms benign, malignant and unspecified (incl cysts and polyps) | Malignant neoplasm progression | 1,092 | 33.06 (30.97-35.29) | 28.12 (28024.02) | 4.74 (4.65) | 27.46 (25.73) |
|  | Ovarian cancer recurrent | 116 | 306.44 (249.34-376.62) | 301.44 (27411.31) | 6.29 (5.99) | 238.07 (193.71) |
|  | Recurrent cancer | 35 | 23.43 (16.75-32.77) | 23.32 (732.56) | 3.79 (3.30) | 22.86 (16.35) |
|  | Myelodysplastic syndrome | 16 | 3.80 (2.33-6.21) | 3.79 (32.84) | 1.61 (0.89) | 3.79 (2.32) |
|  | Metastases to central nervous system | 14 | 3.46 (2.05-5.85) | 3.45 (24.35) | 1.47 (0.69) | 3.45 (2.04) |
|  | Neoplasm | 14 | 3.80 (2.25-6.43) | 3.80 (28.76) | 1.58 (0.80) | 3.79 (2.24) |
|  | Ovarian cancer metastatic | 12 | 66.45 (37.11-119.00) | 66.34 (729.39) | 3.33 (2.48) | 62.71 (35.02) |
|  | Abdominal neoplasm | 7 | 23.24 (10.99-49.14) | 23.22 (145.84) | 2.42 (1.32) | 22.77 (10.77) |
|  | Malignant pleural effusion | 6 | 10.91 (4.88-24.38) | 10.90 (53.42) | 1.95 (0.76) | 10.80 (4.83) |
|  | Cancer pain | 5 | 4.90 (2.03-11.79) | 4.89 (15.43) | 1.30 (0.01) | 4.88 (2.03) |
|  | Hepatic neoplasm | 5 | 5.05 (2.1-12.16) | 5.05 (16.16) | 1.33 (0.03) | 5.03 (2.09) |
| Surgical and medical procedures | Surgery | 49 | 2.81 (2.12-3.72) | 2.79 (56.49) | 1.40 (0.99) | 2.79 (2.11) |
|  | Transfusion | 31 | 8.30 (5.82-11.83) | 8.27 (196.7) | 2.70 (2.18) | 8.21 (5.76) |
|  | Radiotherapy | 7 | 14.24 (6.75-30.02) | 14.22 (85.00) | 2.22 (1.13) | 14.06 (6.67) |
|  | Fluid replacement | 4 | 42.18 (15.54-114.49) | 42.16 (154.95) | 1.86 (0.40) | 40.68 (14.99) |
|  | Intestinal operation | 4 | 9.96 (3.72-26.67) | 9.96 (31.96) | 1.51 (0.06) | 9.88 (3.69) |

ROR, reporting odds ratio; CI, confidence interval; PRR, proportional reporting ratio; χ^2^, chi-squared; IC, information component; EBGM, empirical Bayesian geometric mean.
